# Development and Evaluation of a Radiomics-Based 3D Volumetric and Densitometric Tomographic Scoring System for Chronic Rhinosinusitis with Nasal Polyposis: A Comparative Analysis

**DOI:** 10.3390/jpm16050244

**Published:** 2026-04-30

**Authors:** Simonetta Masieri, Elona Begvarfaj, Pasquale Frisina, Carlo Cavaliere, Antonella Loperfido, Francesca Lombardi, Marcella Bugani, Daniela Messineo

**Affiliations:** 1 Department of Oral and Maxillofacial Sciences, Sapienza University of Rome, Piazzale Aldo Moro 5, 00185 Rome, Italy; s.masieri55@gmail.com; 2 Department of Sense Organs, Sapienza University of Rome, Piazzale Aldo Moro 5, 00185 Rome, Italy; lombardi.1787344@studenti.uniroma1.it (F.L.); marcella.bugani@gmail.com (M.B.); 3 Department of Radiological, Oncological, and Anatomo-Pathological Sciences, Sapienza University of Rome, Piazzale Aldo Moro 5, 00185 Rome, Italy; 4 Department of Life Sciences, Health and Health Professions, Link Campus University, Via del Casale di S.Pio V 44, 00165 Rome, Italy; 5 Otolaryngology Unit, San Camillo Forlanini Hospital, Circonvallazione Gianicolense 87, 00152 Rome, Italy; aloperfido@scamilloforlanini.rm.it

**Keywords:** CRSwNP, radiomics, monoclonal antibodies, computer-assisted image analysis, Lund-Mackay

## Abstract

**Background/Objectives**: The therapeutic effectiveness of chronic rhinosinusitis with nasal polyposis (CRSwNP) depends on an accurate diagnosis that identifies disease characteristics, evaluates sinus patency, and detects paranasal sinus obliteration. This study aims to assess a novel artificial intelligence (AI) system integrated with radiomic analysis for the radiological evaluation of CRSwNP, developing a reliable and predictive clinical-radiological scoring system. **Methods**: This study retrospectively evaluates CT scans of patients with CRSwNP. Image analysis was performed using Radiomica LifeX (Local Image Features Extraction) version 7.5. The extracted densitometric volumes were compared to the Lund-Mackay Score (LMS) to develop a novel scoring system (P-ABCD score) and assess its radiomic predictive capability. **Results**: Twenty patients with CRSwNP undergoing Dupilumab therapy participated in this study. The P-ABCD score, derived from sinus CT imaging data, served as a valuable objective measure of clinical improvement following CRSwNP treatment. **Conclusions**: Advanced radiomic imaging techniques of the sinus cavity provide precise volumetric data combined with texture analysis. These techniques offer high sensitivity by accurately quantifying the true extent of inflammatory involvement in the paranasal sinuses, enabling effective disease stratification.

## 1. Introduction

Chronic rhinosinusitis (CRS) is a chronic inflammatory condition of the nose and paranasal sinuses [1] that affects 5–12% of the world population [2,3,4,5]. Symptoms of CRS include anterior or posterior nasal discharge, nasal obstruction, loss of smell, and/or facial pressure for more than 12 weeks [1,6]. The accurate diagnosis of CRS is based on clinical symptoms, nasal endoscopy findings, and paranasal sinuses imaging [6]. CRS is classified into two main phenotypes: CRS with nasal polyps (CRSwNP) and CRS without nasal polyps (CRSsNP) [6]. CRSwNP affects 1–2% of the European population [7] and is associated with more severe symptoms, greater morbidity, recurrence rate, and increased asthma severity [5,8,9,10].

Computed tomography (CT) is a highly effective radiological tool for detecting and localizing sinus opacifications. It provides detailed anatomical information on the paranasal sinuses, including variations associated with CRS, such as nasal septal deviation and concha bullosa [11,12], and is also essential prior to sinus endoscopic surgery. CT is commonly used as an objective diagnostic tool for the differential diagnosis of sinonasal pathologies and to assess the severity of CRSwNP [13]. The principal findings suggesting a diagnosis of CRS include mucosal thickening and partial or complete opacification of the paranasal sinuses with a homogeneous soft-tissue mass [14,15]. Unlike standard X-rays, CT provides objective information on the extent of sinus disease and is considered the gold standard for staging CRSwNP [16]. Several staging systems based on radiographic grading scales, level of mucosal thickness, 3-D volumetric analysis, and opacification rates have been proposed to increase the diagnostic and prognostic values of CT scans [17,18,19]. Among these, the Lund-Mackay score (LMS) [20] (Table S1) is the most widely used method for radiographic grading of CRSwNP, due to its ease of use and high interobserver reliability [21]. Its use does not require particular training and is explicit. The LMS scores each paranasal sinus individually on a scale from 0 to 2 on each side (0, no opacification; 1, partial opacification; 2, total opacification). The scoring of the ostiomeatal complex (OMC) is either 0 (patent) or 2 (obstructed). The total is the sum of both sides. However, any opacification from ‘none’ to ‘total’ is scored as 1, limiting disease stratification.

The comparative study by Zinreich highlighted that the LMS does not account for the volume of sinus inflammation. As a result, a modified version of the LMS (LMS modified) was proposed to further stratify the level of inflammation [19] (Table S2). Zinreich divided the level of opacification into intervals of 25%, and scored it on a scale from 0 to 5 for every sinus on each side. The OMC score remains the same as LMS.

Several studies have utilized CT as an outcome measurement tool to assess the effectiveness of oral and topical glucocorticoids, amphotericin B, and capsaicin [22,23]. In the era of biologic treatments for severe CRSwNP, imaging can serve as an objective tool to assess treatment efficacy [24,25,26,27]. A major limitation of the current staging systems is their potential to either overestimate or underestimate the degree of sinus inflammatory involvement, which can have significant implications for diagnosis, therapy, and prognosis.

Current conventional radiological assessment relies on a semiquantitative “eyemetric” estimate of sinus opacification, in which the radiologist assigns discrete scores of 0–1–2 based on visual perception of opacity. LMS and its modified version can assign the same score to different categories or assign different scores to similar categories, not permitting a personalized and precise diagnosis of the extent and gravity of the disease. For example, total opacification of paranasal sinuses is scored as 2 or 100%, while opacification of 99% is scored as 1 or 4, showing inconsistency in the scoring logic. The radiomics approach enables a quantitative, standardized three-dimensional assessment.

This is a preliminary study that aims to evaluate a new AI system combined with radiomic analysis to develop a clinical-radiological score based on precise measurement of inflammatory tissue in the paranasal sinuses. Although the new system does not use machine learning or deep learning algorithms, it represents an advanced form of computer-assisted radiomics analysis, automating segmentation and parameter extraction, thereby significantly reducing operator subjectivity. The system aims for a personalized diagnosis because the analysis of the sinus opacification is based on clear anatomical boundaries, which are manually delineated on a case-by-case basis. Personalized diagnosis makes the most accurate and bespoke treatment possible.

## 2. Materials and Methods

This is a retrospective single-center real-life observational study conducted at the Rhinoallergology Units of the Otolaryngology-Head and Neck Surgery of Azienda Ospedaliera Roma 1 (Sapienza University of Rome and San Camillo Forlanini Hospital), Rome, Italy. The study included patients receiving dupilumab treatment for CRSwNP that was not controlled by standard therapies between January 2023 and December 2024, in accordance with the AIFA (Agenzia Italiana del Farmaco) guidelines and the EPOS/EUFOREA update [7,28].

Exclusion criteria for dupilumab treatment included minors under 18 years of age, pregnant or lactating women, patients currently undergoing other biologic or immunosuppressive therapies, oncologic patients receiving adjuvant therapy or who had undergone radiotherapy or chemotherapy in the past twelve months, and patients with chronic autoimmune conditions. Additionally, patients who did not consent to share their clinical information were excluded from the study. Ethics committee approval was obtained, and informed consent for the use of clinical data and privacy protection was acquired from all patients before the initiation of therapy.

Patients were assessed at baseline (T0) before starting dupilumab and followed up every six months, until a period of 24 months (T1). To objectively assess the presence of CRSwNP and the extent of nasal polyposis, each patient underwent a comprehensive ENT physical examination, including nasal endoscopy and a sinus CT scan. The nasal endoscopy assessed the presence of nasal polyps and, therefore, the severity of the nasal polyposis via the Nasal Polyps Score (NPS) [7]. The CT scan was used to evaluate the extent of sinonasal disease prior to treatment initiation and after a period of 24 months of therapy. Symptom severity and health-related quality of life (QoL) were measured using the sum of the Visual Analog Scale (VAS) for the following symptoms: nasal obstruction, rhinorrhea, anosmia, and post-nasal drip, and the SNOT-22 questionnaire [29]. The Asthma Control Test (ACT) was used to evaluate asthma control (scores over 21 indicating well-controlled asthma). Dupilumab was an add-on treatment, as all the patients continued the standard therapy for CRSwNP with nasal rinses and intranasal corticosteroid.

CT scans of twenty patients were analyzed before and after 24 months of dupilumab therapy, and scored according to the LMS and the modified LMS. Since the comparison focused on applying different scoring systems to the same group of patients pre- and post-treatment, patients served as their own paired controls in a before-and-after design. For this study, we evaluated the CT scans using a high-resolution volumetric HRCT scanner (CT Aquilion, Toshiba, 16 channels, Tokyo, Japan), with a slice width of 0.5 mm. The scan parameters were adjusted according to the patient’s characteristics, ranging from 120 to 100 kV and 100–50 mA, with a rotation time of 1–0.5 s. Multiplanar reconstruction (MPR) was performed with an approximate dose of 8 mGy. Radiographic tomographic evaluation was performed using standard multiplanar reconstruction and anatomical landmarks. Image analysis was conducted with the Radiomica LifeX software (Local Image Features Extraction; Institut Curie, Paris, France) version 7.5, designed for texture analysis of DICOM images. The images were imported into the Lifex platform and saved as DCM PatientDPI files.

Radiomic feature extraction from tomographic images in this study was performed by calculating the actual sinus volumes. This was achieved by plotting a 3D volumetric region of interest (ROI) using tomographic navigation and the circle function, which defines a reconstruction volume (VOI). The resulting data was saved in the DCM R1.rtstruct format. The following cephalometric points were used as the boundaries of the primary navigation ROI: S, central point of the sella turcica; N, located at the level of the frontonasal suture; Ba, anterior limit of the foramen magnum; ANS, tip of the anterior nasal spine; PNS, posterior boundary of the hard palate; A, located on the maxillary symphysis, just below the anterior nasal spine; MX, left and right, the deepest points between the lateral wall of the maxillary bone and the lower edge of the maxillary process of the zygomatic bone.

**Figure 1:**
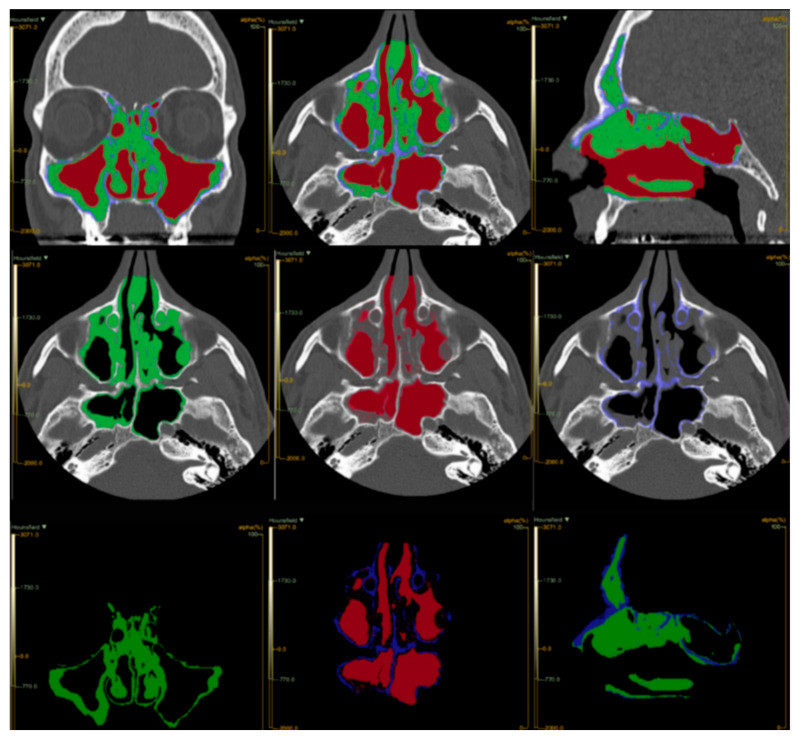
The figure illustrates distinct ROIs within the defined radiodensity range (−2000 to +2000 HU), segmented for volumetric analysis. Volumetric processing enables the differentiation of bone (blue), inflammatory tissue (green), and air (red).

We defined the VOI using the Lifex nROI function (Figure 1). The VOI values were automatically scaled according to the densitometric reference values of the sub-nROI for air, soft tissue, and bone, establishing the following Hounsfield Unit (HU) ranges: −2000 to −250 for air, −249 to +250 for soft tissue, and +251 to +2000 for bone. The software automatically saved the extracted characteristics in R1_Abs_thes format within the “ROI-Value” folder in DCM format. This enabled an initial analysis of the radiomic texture of the images by generating a histogram of voxel distribution per UH unit and calculating the total volumes (cubic centimeters: cm^3^) of air and soft tissue. Additionally, the Lifex artificial intelligence algorithm computed the maximum, minimum, and average HU values.

The volumes of interest (VOI) for the ROIs and sub-ROIs were extracted using LifeX’s automated image calculation algorithm, measured in cm^3^, as follows: Total Vol (the total volume calculated for each patient), Soft Vol (the volume occupied by soft tissue), Air Vol (the volume of air detected), and % Air and % Soft (the relative percentage of each component compared to the total). The obtained densitometric volumes were compared with the LMS and the modified LMS to assess radiomic predictability.

A percentage-based analysis of the radiomic images enabled the definition of a novel radiological and clinical index. This new score, P(ABCD), was calculated as the percentage of volumetric soft-tissue texture within the range of −249 to +250 HU, relative to the absolute patency value, by the following formula: P(ABCD) = V_soft_/(V_soft_ + V_air_), where V_soft_ represents the volume of soft tissue within the Hounsfield Unit range −249 to +250 HU, and V_air_ represents the air volume within the segmented sinus cavity. The computational workflow consisted of: (1) segmentation of the sinus volume of interest (VOI); (2) voxel classification based on HU thresholds; (3) extraction of volumetric parameters (V_soft_ and V_air_), and (4) calculation of the P(ABCD) ratio.

The VOI of soft tissues was considered 100% as the reference maximum. The score ranges from 0 to 1, with six defined grades: P0 (Absence of inflammation): 0; P1 (Pansinusitis): 0.9–1; A (Mild): 0–0.15; B (Moderate): 0.15–0.35; C (Advanced): 0.35–0.55; D (Severe): 0.55–0.9.

**Figure 2:**
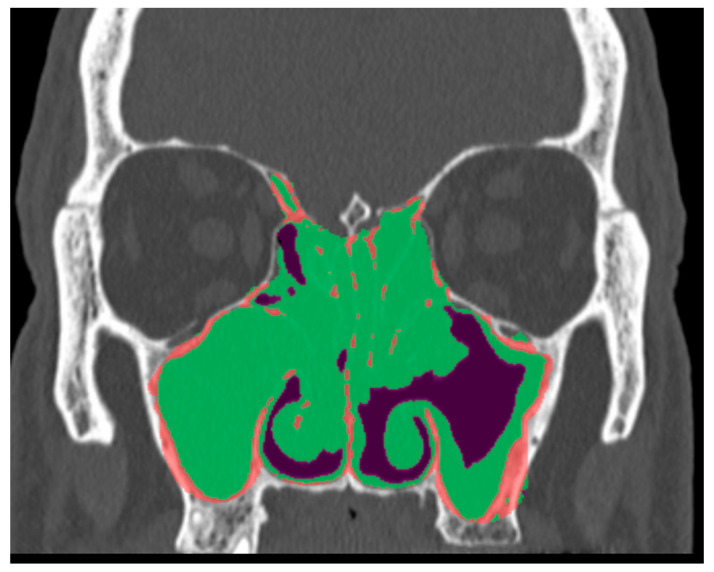
The figure illustrates distinct ROIs within the OMC. Volumetric processing enables the differentiation of bone (red), inflammatory tissue (green), and air (purple). Right, 2 (obstructed); Left, 0 (patent).

If the OCM was obliterated, a score ranging from 0 (patent) to 2 (obstructed) was assigned based on the degree of OMC involvement (Figure 2). This scoring applied equally to patients regardless of surgical history. This criterion was established because the obliteration of the OMC by inflammatory tissue has a direct incremental effect on the soft tissue VOI.

The sample was analyzed using descriptive statistics, with means and standard deviations (SD) calculated for quantitative variables. Results were summarized in graphs and tables. Differences between means were assessed using Friedman and Wilcoxon nonparametric tests, with corrections for multiple comparisons. A significance level of *p* < 0.05 was applied. Multiple comparisons were corrected using the Bonferroni method following non-parametric statistical testing. All analyses were performed using STATA software vers.13 (StataCorp Release 13. College Station, TX, USA). To ensure reproducibility, all segmentation parameters, Hounsfield Unit thresholds, and computational steps have been explicitly defined.

All quantitative image analyses, statistical plots, and radiomic comparisons were performed in a Python 3.10 environment, available upon request, using standard scientific and visualization libraries:

- NumPy (v1.26)—for numerical computation and array-based data processing (calculation of mean, standard deviation, and confidence limits);
- pandas (v2.2)—for structured tabular data management;
- Matplotlib (v3.9)—for generation of graphical outputs, including Bland–Altman and ROC plots;
- scikit-learn (v1.5)—for advanced statistical analysis, ROC curve computation, and AUC estimation;
- SciPy (v1.13)—for supporting inferential statistics (normality testing and correlation analysis).

The Bland–Altman plots (Figures S1 and S2) were generated using these libraries to compute the mean difference (bias) and limits of agreement (±1.96 SD) between methods. All calculations were executed on anonymized datasets using internally developed scripts, and the complete codebase is available to the journal and reviewers upon request.

## 3. Results

A total of 20 patients were included, 11 males (55%) and 9 females (45%). The mean age was 66.04 years (20–88 years). The mean body mass index (BMI) was 25.17 (19.1–38.7), indicating a slight overweight status in the cohort. Among our patients, 40% suffered from concomitant asthma, with good control of the disease (ACT at the baseline was 21). In 65% of cases, we found evidence of concomitant allergies, especially for dust mites (*Dermatophagoides farinae* and *Dermatophagoides pteronyssinus*), grasses, and Parietaria; 15.3% of patients had intolerance to Non-Steroidal Anti-Inflammatory Drugs (NSAIDs). The allergic patients did not undergo concomitant allergen immunotherapy. In our series, all of the patients underwent at least one surgery before starting dupilumab: in 42.4% of cases, it was only a single functional endoscopic sinus surgery (FESS) procedure before starting biologic therapy, whereas in 40.8% of cases, dupilumab was the therapeutic choice after two or more FESS procedures. All the surgeries were carried out at the same centers by the same surgeons. The range of surgeries performed before biological therapy was 1 to 6. At baseline, the mean values for NPS, the SNOT-22 questionnaire, and total VAS were 4.14, 45.1, and 32.67, respectively. A summary of all the described anamnestic features of patients can be found in Table 1.

All the asthmatic patients had controlled asthma, which showed further improvement after the therapy with dupilumab. There was an overall improvement of the CRS-related symptoms, evidenced by the decrease in the VAS and SNOT-22 scores from baseline to 24 months of treatment, from 32.67 (12.27) to 10.95 (1.45), and from 45.1 (19.73) to 16.48 (13.27), respectively. A significant reduction in polyp size was observed, as evaluated by nasal endoscopy, decreasing from 4.14 (1.04) at T0 to 0.75 (0.81) at T1. A summary of the clinical outcomes prior to and following therapy with dupilumab is presented in Table 2.

The data presented in Table 3 illustrate the trends in the main radiological parameters assessed by CT and analyzed with LIFEx software before and after dupilumab administration. As shown in the table, all parameters considered (LMS, Modified LMS, and P(ABCD)) showed significant reductions in mean and median values after treatment, with statistically significant differences (*p* < 0.05 or *p* < 0.01). These results confirm the efficacy of dupilumab in reducing sinus opacification, as assessed using different radiological scoring systems.

Table 4 summarizes the main results from the analysis of volumetric and densitometric parameters extracted with the LIFEx software, comparing data collected before the start of dupilumab therapy and after 24 months of treatment. The values reported represent the average calculated from the 20 patients included in the study. The analysis shows a reduction in total and soft-tissue volumes, while air volume and the percentage of air relative to total volume increase. Concurrently, the rate of soft tissue decreases from the initial value.

Table S3 reports the volumetric and densitometric parameter values extracted using the LIFEx software for each patient, both pre-treatment and after 24 months of dupilumab therapy, allowing for direct verification of the changes observed in each patient. The data are organized in pairs for each case, allowing for observation of any changes in the parameters after treatment.

The three methods generated significantly different results in measuring the extent of sinus opacification. In particular, the measurement obtained using the P(ABCD) score (62.2%) was, on average, considerably higher than those obtained using the modified LMS (39.3%) and the LMS (45.3%). Table 5 displays the mean, standard deviation, minimum, and maximum values of the sinus opacification measured with the three methods.

**Figure 3:**
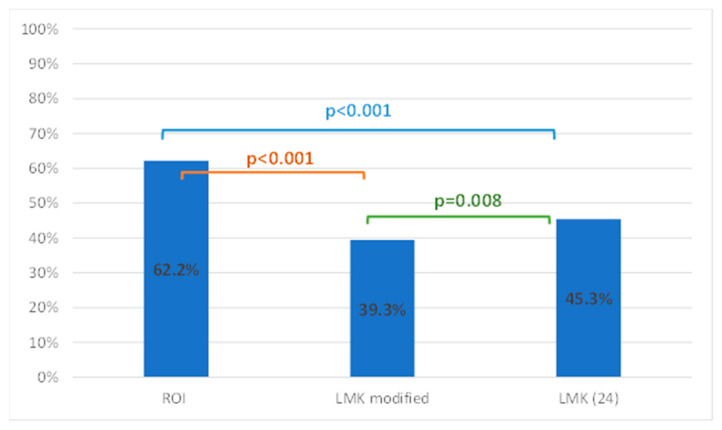
Correlation between P(ABCD), LMS, and LMS modified.

The differences among the three methods were evaluated using the nonparametric Friedman test, without assuming normality. Pairwise comparisons were then performed using the Bonferroni post hoc test, with multiple comparisons corrections (Figure 3).

**Figure 4:**
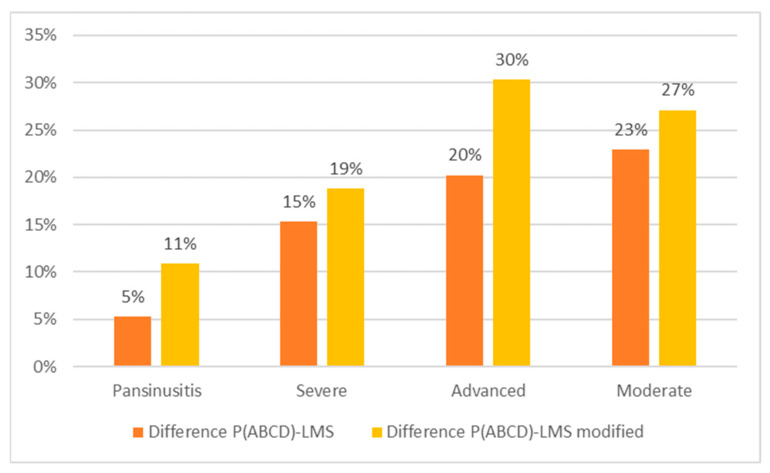
Differences between the P(ABCD) score and LMS, and the P(ABCD) score and LMS modified.

To assess whether LMS and LMS modified scores underestimate compared to the P(ABCD), we categorized the scores into six grades: P0 (absence) = 0; P1 (pansinusitis) = 0.9 or 1; Mild A = 0 to 0.15; Moderate B = 0.15 to 0.35; Advanced C = 0.35 to 0.55; and Severe D = 0.55 to 0.9. Patients were categorized as Pansinusitis, Severe sinusitis, Advanced and Moderate sinusitis, based on the P(ABCD) score. We calculated average scores for P(ABCD), LMS, and modified LMS for each group, then compared these results. As shown in Table 6 and Figure 4, the average P(ABCD) score was consistently higher than that of LMS and modified LMS across all subgroups. In the Severe and Advanced groups, differences ranged from 15% to 30% and were statistically significant given the sample sizes (n = 20 and n = 14). No subjects were classified as Mild.

Figure 4 demonstrates that the discrepancies between the P(ABCD) score and the other two methods increased as the opacity grade decreased. In particular, the underestimation relative to the P(ABCD) score was more pronounced for the modified LMS. To quantitatively document the stability and consistency of P(ABCD) compared to LMS, we also performed a Bland–Altman analysis (Figures S1 and S2). Since pre- and post-treatment measurements were obtained from the same subjects, the paired nature of the data was considered in the interpretation of the Bland–Altman analysis.

## 4. Discussion

The need for quality staging systems to assess the severity and treatment response in CRSwNP is not new; it dates back to 1893 [30,31]. A century later, Kennedy proposed that staging systems could influence surgical outcomes by assessing the extent of the disease [32]. In 1993, Lund and Mackay introduced the eponymous staging system as a simple tool to guide the surgical treatment of the CRSwNP [20]. Several scoring systems exist to assess rhinosinusitis status [12,14,15,17,18], and their ability to standardize disease severity has enabled objective treatment interventions and outcomes analysis. The systematic review of Greguric et al. [33] identified the LMS as the most suitable system for clinical practice, based on its ease of use, after analyzing four rhinosinusitis CT scoring systems: Jorgensen, May and Levine, Lund-Mackay, and Newman.

The Task Force on Rhinosinusitis [34] later recommended the LMS as the reference system for future outcome research. Zinreich observed that although the Lund-Mackay system is widely accepted, objective, and reproducible, it does not provide detailed information on the volume of inflammatory disease [19]. He proposed a modified version to improve disease stratification. Chen’s review [35] found that among various rhinosinusitis CT staging systems, only the LMS and Zinreich’s modified LMS were most frequently used and suitable for meta-regression, recognizing their criticalities.

In this study, we evaluated sinus opacification and its changes in response to dupilumab treatment in CRSwNP patients, using CT scans. Although the sample may be subject to bias, it enabled precise and timely follow-up. We compared the widely used LMS scoring system and its modified version, which offers improved disease stratification, with a new objective computerized method, the P(ABCD) score. The objective was to assess how these systems correlate in defining disease severity, as measured by paranasal sinus opacification, and how this changes with treatment. While endoscopic findings are important for evaluating nasal blockage, CT scans are more effective for determining the extent of CRSwNP in the paranasal sinuses.

Dupilumab was a safe, well-tolerated, and effective treatment for patients affected by CRSwNP. No adverse effects from the treatment were detected during the study. All the patients demonstrated a significant improvement of the CRS-related symptoms, as confirmed by the reduction in the mean values of SNOT-22 and VAS score compared to the baseline. The rise in the ACT score from the baseline was indicative of the effect of dupilumab in asthma control. As shown in Table 2, the greatest effect of dupilumab was the reduction in polyp size. Our results are consistent with the data from previous studies [16,24,25,27].

All three scoring systems showed significant reductions in the mean and median values of sinus opacification after treatment, with statistically significant differences (*p* < 0.05 or *p* < 0.01). These results confirm the efficacy of dupilumab in reducing sinus opacification, as assessed using different radiological scoring systems.

The analysis shows a reduction in total and soft-tissue volumes, while air volume and the percentage of air relative to total volume increase. These changes suggest a positive effect of dupilumab therapy on volumetric remodeling assessed by radiomic imaging. Overall, reductions in both total and soft-tissue volumes were observed, accompanied by increases in air volume and air percentages, suggesting a possible reduction in edema or inflammation following therapy. For example, in case 1, the total volume increased slightly after treatment (from 106,893 to 109,033 cm^3^), while the percentage of air increased from 13% to 24%, and the rate of soft tissue decreased from 87% to 76%. In case 8, a marked increase in the percentage of air (from 26% to 69%) and a reduction in the rate of soft tissue (from 74% to 31%) were observed (Table S3).

These changes are indicative of therapy-induced structural changes and will be the subject of further investigation.

In our study, the “objectivity” of the P(ABCD) score refers not to a single diagnostic metric, but to the computational, three-dimensional, and not entirely operator-independent nature of the measurement process. Unlike the semiquantitative Lund–Mackay and modified LMS systems, which rely on discrete visual assessments and are inherently operator-dependent, the P(ABCD) is derived from automatic voxel-based segmentation and the extraction of continuous volumetric and densitometric parameters (cm^3^ and HU) of sinus soft tissue. This approach minimizes inter- and intra-observer variability; avoids the “category jumps” typical of ordinal scales, and provides a proportional and reproducible measure of inflammatory burden.

Both Bland–Altman analyses confirm that the P(ABCD) score produces consistent but biased higher estimates than the Lund–Mackay systems, consistent with its three-dimensional, voxel-based nature. The narrow limits of agreement and the absence of a mean-dependent trend demonstrate the systematic stability and reproducibility of the radiomics method. The comparison with the modified LMS (Figure S2) is clinically more representative. At the same time, the classic LMS (Figure S1) highlights the conceptual leap between a discrete visual assessment and a continuous quantitative measurement. The analysis was conducted using the percentage values of sinus opacification/permeability derived from three corresponding datasets: the ROI-derived P(ABCD) values obtained from volumetric segmentation and densitometric analysis through LIFEx; the LMS percentages calculated from the traditional point system; and the modified LMS percentages derived from its expanded grading system.

The P(ABCD) score systematically yields higher values than both LMS and LMS modified, with mean biases of +0.17 and +0.23, respectively. This confirms the greater sensitivity and objectivity of the radiomic method in detecting residual inflammatory tissue volume, while maintaining consistent variability across the measurement range. The limits of agreement are narrow (−0.007 to +0.34 for LMS; +0.01 to +0.44 for LMS modified), demonstrating a stable and predictable relationship between the compared methods. Considering the Lund–Mackay score as a widely accepted clinical reference standard, the deviation of P(ABCD) from LMS highlights the increased sensitivity of the volumetric radiomic approach, particularly in intermediate stages of disease.

This study found that the P(ABCD) score, calculated from sinus CT image data, is a potential objective measure correlated with the reduction in the sinus opacities after CRSwNP therapy. The Lund-Mackay scoring system also correlated with improvement after dupilumab therapy. However, the stronger correlation of the P(ABCD) score with changes in objective data highlights the greater effectiveness of accurately measuring the volume of soft tissues in the paranasal sinuses. This approach is more precise than using a scoring system that assigns the same score (e.g., “1”) to represent a broad range of mucosal involvement, from 1% to 99%, for any given sinus. In evaluating patients’ CT scans both before and after treatment, we not only graded the level of sinus opacification but also measured the density and exact percentage of opacity in each sinus using radiographic HU and the ratio of soft tissue to air. Once the sinus borders were outlined, the algorithm enabled precise calculation of the total volume, air volume, and disease volume. By distinguishing different Hounsfield units, the P(ABCD) score can identify the percentage of disease volume in each sinus, and the mean of these values represents the total disease volume for that patient.

A strength of our study is the accurate calculation of the total volume of the paranasal sinuses, achieved through precise anatomical landmarks, and an accurate ratio between soft tissue and air within the paranasal cavities, which leads to a unique and very personalized diagnosis. Additionally, the use of AI systems for all calculations eliminated the risk of human errors. AI analysis systems have recently gained attention as tools to improve medical performance [36]. Still, like all computerized methods, a key limitation of this study is related to software availability. Though, because of the rapid development of AI systems, we are confident that soon even the non-tertiary healthcare setting will be provided with sophisticated resources. The manual delineation of sinus boundaries is another primary limitation, as it could introduce subjectivity. Any deviation from the specified landmarks could lead to inaccurate disease percentage scores. Additionally, the time required to learn and perform the ROI calculation technique presents another limitation. However, due to the inter-individual variability of the anatomical landmarks, before and after sinus surgery, the manual VOI delineation is mandatory.

With the development of an automated, more time-efficient, and user-friendly system, we are confident that the P(ABCD) score will become a useful instrument for assessing the disease extent, even for other CRS populations.

## 5. Conclusions

AI-derived CT analysis offers an objective and reproducible method for measuring disease extent in CRSwNP and is sensitive to changes in sinus opacification resulting from therapeutic interventions. The P(ABCD) score provides a comprehensive and objective approach to assessing disease severity and treatment efficacy in patients with CRSwNP. However, its adoption may be hindered by its complexity and limited accessibility.

## Figures and Tables

**Table 1:** Demographic and clinical data at baseline.

Age in Years (Mean; SD)	66.04 (12.71)
Sex (n; %)	F: 9 (45%) M: 11 (55%)
BMI kg/m^2^ (mean; SD)	25.17 (4.42)
Number of surgeries for patient (mean; SD)	1.81 (1.71)
Atopics (n; %)	13 (65%)
Asthma (n; %)	8 (40%)
Nasal Polyps score (n; %)	4.14 (1.04)
SNOT-22 (n; %)	45.1 (19.73)
Total VAS (n; %)	32.67 (12.27)
ACT (n; %)	21 (4.5)

**Table 2:** Clinical outcomes before (T0) and after (T1) therapy with dupilumab.

	T0	T1	T1-T0 (%)
Mean SNOT-22 Std. dev	45.1 19.73	16.48 13.27	−63.00
Mean VAS Std. dev	32.67 12.27	10.95 8.46	−66.00
Mean ACT Std. dev	21 4.5	24 1.8	+14.29
Mean NPS Std. dev	4.14 1.04	0.75 0.81	−82.00

**Table 3:** Paranasal opacifications before and after dupilumab therapy, scored by LMS, Modified LMS, and P(ABCD), expressed in mean and median values.

Scoring System	Mean (Pre-Treatment)	Mean (Post-Treatment)	Δ (Post–Pre-Treatment)	Median (Pre-Treatment)	Median (Post-Treatment)	Wilcoxon *p*-Value
LMS	8.7	6.1	−2.6	8	6	0.031 *
Modified LMS	7.9	5.8	−2.1	8	6	0.028 *
P(ABCD)	0.68	0.52	−0.16	0.70	0.51	0.009 **

* *p* < 0.05; ** *p* < 0.01.

**Table 4:** Analysis of volumetric and densitometric parameters extracted with the LIFEx software before and after 24 months of treatment.

Parameters	Mean Pre-Treatment	Mean Post-Treatment	Δ (Post–Pre-Treatment)
Vol Tot (cm^3^)	118,000	110,000	−8000
Vol Soft (cm^3^)	85,000	70,000	−15,000
Vol Air (cm^3^)	25,000	40,000	+15,000
% Air (%)	25%	36%	+11%
% Soft (%)	75%	64%	−11%

**Table 5:** Mean, standard deviation, minimum, and maximum values were for the P(ABCD) score, LMS, and LMS modified.

Staging System	Mean	Std. Deviation	Minimum	Maximum	Friedman Test *p*-Value
P(ABCD) score	62.2%	20.1%	28.0%	96.0%	<0.001
LMS modified	39.3%	28.0%	2.0%	91.0%	
LMS	45.3%	26.2%	4.0%	92.0%	

**Table 6:** Mean, standard deviation, minimum, and maximum values were measured for the three different staging systems: LMS, LMS modified, and P(ABCD) for any subgroup of rhinosinusitis.

		P(ABCD)	LMS	LMS Mod.	Difference P(ABCD)-LMS	*p*-Value	Difference P(ABCD)-LMS Mod.	*p*-Value	n
Pansinusitis	Mean	93.6%	82.7%	88.3%	5.3%	0.109	10.9%	0.109	3
	Std.dev	1.9%	7.5%	3.1%	3.8%		7.8%		
Severe	Mean	74.1%	55.3%	58.7%	15.3%	<0.001	18.8%	<0.001	20
	Std. Dev.	11.1%	18.3%	18.1%	9.9%		11.1%		
Advanced	Mean	45.2%	14.9%	25.0%	20.2%	<0.001	30.3%	<0.001	14
	Std. Dev	6.9%	9.4%	10.1%	6.0%		7.4%		
Moderate	Mean	30.5%	3.3%	7.6%	22.9%	0.109	27.1%	0.109	3
	Std. Dev	2.1%	1.3%	4.4%	6.4%		0.9%		
Mild	Mean								0
	Std. Dev								

## Data Availability

Data are available upon request to the corresponding author.

## Supplementary Materials

The following supporting information can be downloaded at: https://www.mdpi.com/article/10.3390/jpm16050244/s1, Table S1: The Lund-Mackay staging system score; Table S2: The Lund-Mackay modified score by Zinreich; Table S3: Individual volumetric data pre- and post-treatment with dupilumab; Figure S1: Comparison between the P (ABCD) score and the LMS; Figure S2: Comparison between the P(ABCD) score and the modified LMS.
