# Supplementary material for: Development and Evaluation of a Radiomics-Based 3D Volumetric and Densitometric Tomographic Scoring System for Chronic Rhinosinusitis with Nasal Polyposis: A Comparative Analysis
Source: J Pers Med. 2026 Apr 30;16(5):244. doi: 10.3390/jpm16050244 (PMC13208975; doi:10.3390/jpm16050244)
Supplement: Supplementary file 1 [file jpm-16-00244-s001.zip › jpm-4173861-supplementary.pdf]

Table S1. The Lund-Mackay staging system score.

Scores: Each Sinus 0-2, OMC 0 or 2		
**0: Normal, 1: Partial Opacification, 2: Total Opacification**		
**Sinus**	Right	Left
Maxillary		
Anterior ethmoid		
Posterior ethmoid		
Sphenoid		
Frontal		
Osteomeatal complex (OMC)		
Total (maximum 24)		

Table S2. The Lund-Mackay modified score by Zinreich.

Scores: Each Sinus 0-2, OMC 0 or 2		
**0: Normal, 1: Partial Opacification, 2: Total Opacification**		
**Sinus**	Right	Left
Maxillary		
Anterior ethmoid		
Posterior ethmoid		
Sphenoid		
Frontal		
Osteomeatal complex (OMC)		
Total (maximum 24)		

Table S3. Table S3. Individual volumetric data pre- and post-treatment with dupilumab.

Case	Vol Tot (cm^3)	Vol Soft (cm^3)	Vol Air (cm^3)	% Air	% Soft	P(ABCD)
Case 1 pre	106893	92725	14179	13%	87%	0.867456241
Case 1 post	109033	82780	26253	24%	76%	0.277570559
Case 2 pre	141688	114254	27414	19%	81%	0.223044625
Case 2 post	143076	93560	49485	35%	65%	0.39871191
Case 3 pre	124338	108604	16484	13%	87%	0.152830894
Case 3 post	133055	71588	61492	46%	54%	0.5327701
Case 4 pre	117413	96409	21004	18%	82%	0.206223545
Case 4 post	123689	105868	17821	14%	86%	0.855920898
Case 5 pre	77634	47889	29744	38%	62%	0.616856017
Case 5 post	74823	41408	33415	45%	55%	0.616856017

Case 6 pre	79609	54802	24807	31%	69%	0.553412721
Case 6 post	78179	51013	27166	35%	65%	0.652515381
Case 7 pre	96377	51791	44586	46%	54%	0.53737925
Case 7 post	98429	52520	45909	47%	53%	0.533582582
Case 8 pre	91660	68082	23578	26%	74%	0.742766747
Case 8 post	132958	41804	91154	69%	31%	0.314415078
Case 9 pre	154788	81620	73167	47%	53%	0.527301858
Case 9 post	161356	45169	116187	72%	28%	0.279933811
Case 10 pre	104426	90781	13646	13%	87%	0.869333308
Case10 post	105059	81804	23255	22%	78%	0.778648188
Case 11 pre	127180	121762	5418	4%	96%	0.957398962
Case11 post	135505	125134	10371	8%	92%	0.923464079
Case 12 pre	98393	40268	58126	59%	41%	0.355576513
Case12 post	104976	37327	67648	64%	36%	0.355576513
Case 13 pre	76225	65460	10765	14%	86%	0.858773368
Case13 post	67204	33600	33604	50%	50%	0.49997024
Case 14 pre	90519	55586	34933	39%	61%	0.614081022
Case14 post	91072	36713	54359	60%	40%	0.403120608
Case 15 pre	74677	32931	41747	56%	44%	0.44097915
Case15 post	83501	33612	49889	60%	40%	0.92653559
Case16 pre	115607	107114	8493	7%	93%	0.92653559
Case16 post	118315	100328	17966	15%	85%	0.84797363
Case 17 pre	124323	69652	55090	44%	56%	0.560250316
Case17 post	119205	42766	76511	64%	36%	0.358760119
Case 18 pre	92301	29460	62859	68%	32%	0.31917314
Case18 post	92984	34699	58285	63%	37%	0.373171728
Case 19 pre	105180	81064	24133	23%	77%	0.770716866
Case19 post	110227	54467	55788	51%	49%	0.494134831
Case 20 pre	139375	63195	76201	55%	45%	0.45341704
Case20 post	151628	94326	57343	38%	62%	0.622088269

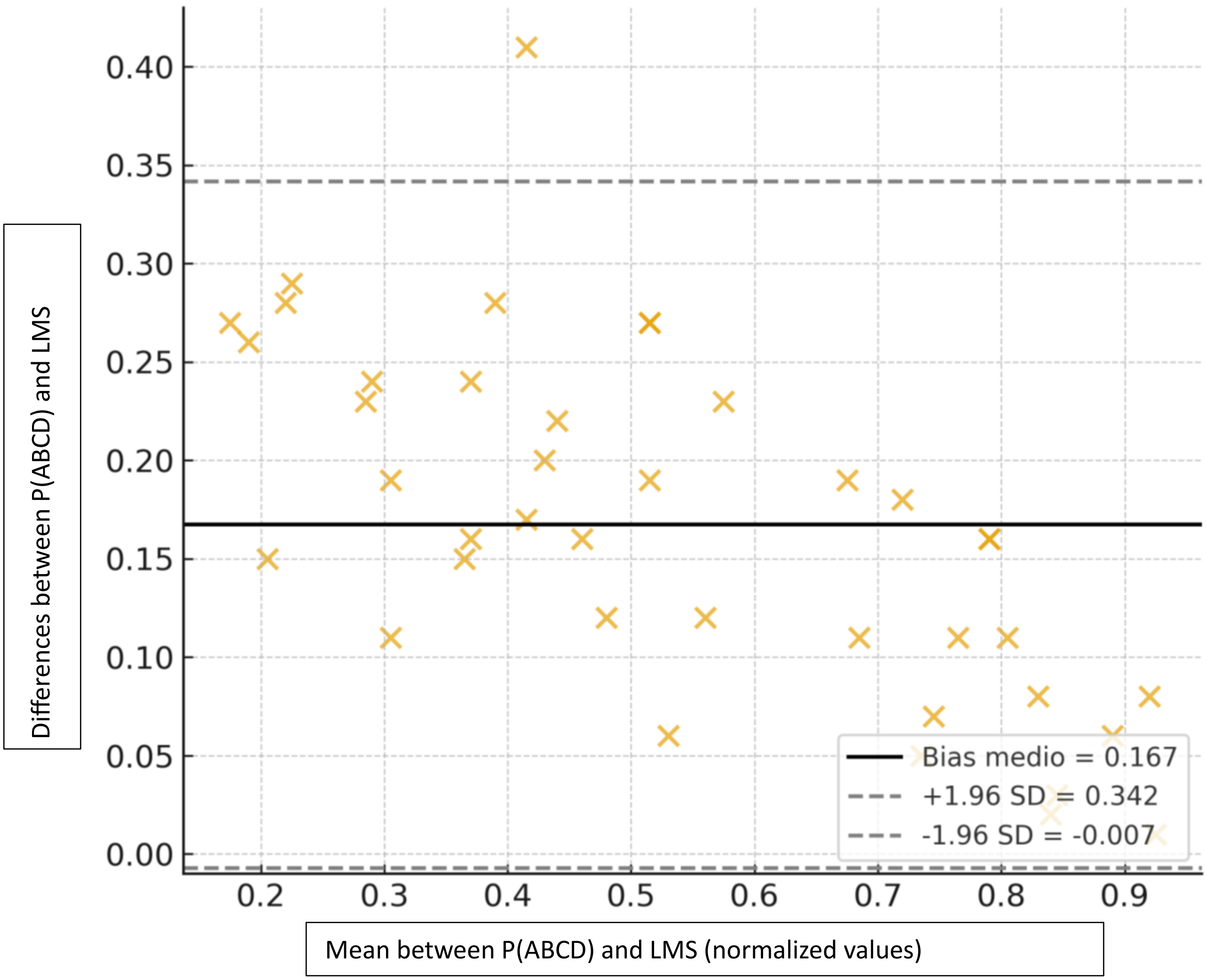

Figure S1. Comparison between the P (ABCD) score and the LMS.

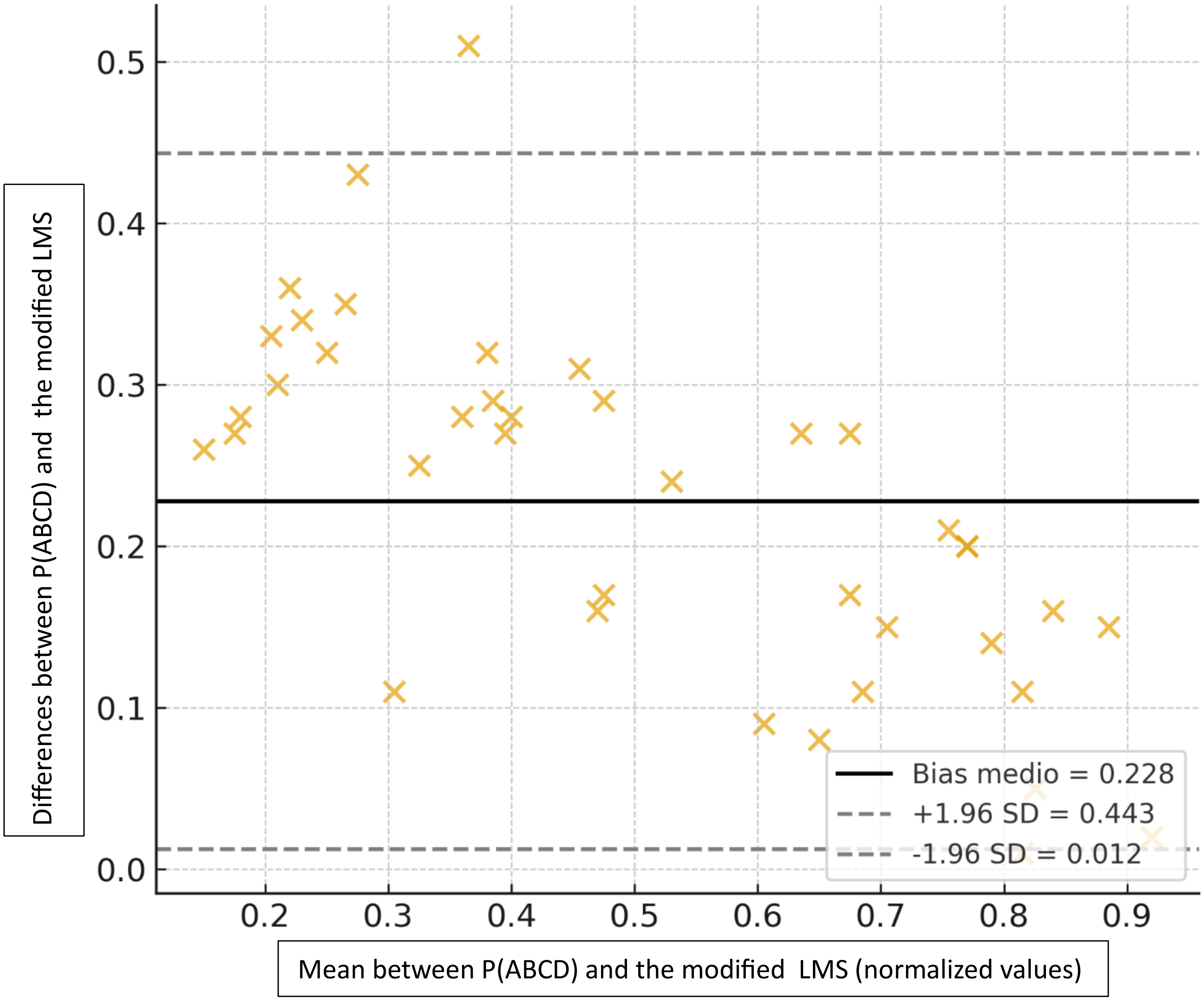

Figure S2. Comparison between the P(ABCD) score and the modified LMS.
